# Inhibition of SARS-CoV-2 Alpha Variant and Murine Noroviruses on Copper-Silver Nanocomposite Surfaces

**DOI:** 10.3390/nano12071037

**Published:** 2022-03-22

**Authors:** Dina A. Mosselhy, Lauri Kareinen, Ilkka Kivistö, Jenni Virtanen, Emil Loikkanen, Yanling Ge, Leena Maunula, Tarja Sironen

**Affiliations:** 1 Department of Virology, Faculty of Medicine, University of Helsinki, 00014 Helsinki, Finland; lauri.kareinen@helsinki.fi (L.K.); ilkka.kivisto@helsinki.fi (I.K.); jenni.me.virtanen@helsinki.fi (J.V.); 2 Department of Veterinary Biosciences, Faculty of Veterinary Medicine, University of Helsinki, 00014 Helsinki, Finland; 3 Department of Food Hygiene and Environmental Health, Faculty of Veterinary Medicine, University of Helsinki, 00014 Helsinki, Finland; emil.loikkanen@helsinki.fi (E.L.); leena.maunula@helsinki.fi (L.M.); 4 VTT Technical Research Center of Finland Ltd., 02044 Espoo, Finland; yanling.ge@vtt.fi

**Keywords:** SARS-CoV-2, Alpha variant, norovirus, copper particles, silver particles, nanocomposites, surface inactivation and application

## Abstract

With the continued scenario of the COVID-19 pandemic, the world is still seeking out-of-the-box solutions to break its transmission cycle and contain the pandemic. There are different transmission routes for viruses, including indirect transmission via surfaces. To this end, we used two relevant viruses in our study. The severe acute respiratory syndrome coronavirus 2 (SARS-CoV-2) causing the pandemic and human norovirus (HuNV), both known to be transmitted via surfaces. Several nanoformulations have shown attempts to inhibit SARS-CoV-2 and other viruses. However, a rigorous, similar inactivation scheme to inactivate the cords of two tedious viruses (SARS-CoV-2 Alpha variant and HuNV) is lacking. The present study demonstrates the inactivation of the SARS-CoV-2 Alpha variant and the decrease in the murine norovirus (MNV, a surrogate to HuNV) load after only one minute of contact to surfaces including copper–silver (Cu–Ag) nanocomposites. We thoroughly examined the physicochemical characteristics of such plated surfaces using diverse microscopy tools and found that Cu was the dominanting element in the tested three different surfaces (~56, ~59, and ~48 wt%, respectively), hence likely playing the major role of Alpha and MNV inactivation followed by the Ag content (~28, ~13, and ~11 wt%, respectively). These findings suggest that the administration of such surfaces within highly congested places (e.g., schools, public transportations, public toilets, and hospital and live-stock reservoirs) could break the SARS-CoV-2 and HuNV transmission. We suggest such an administration after an in-depth examination of the in vitro (especially on skin cells) and in vivo toxicity of the nanocomposite formulations and surfaces while also standardizing the physicochemical parameters, testing protocols, and animal models.

## 1. Introduction

The number of COVID-19 cases reported and updated weekly by the World Health Organization surpasses 370 million confirmed cases and 5.6 million deaths, as of 30 January 2022, with the dominating variants of concerns (VOCs) in descending order being Omicron, Delta, Gamma, Alpha, and Beta [1]. The diverse infection transmission routes of severe acute respiratory syndrome coronavirus 2 (SARS-CoV-2) (through aerosols, droplets, and fomites) adds to the complexity of containing the pandemic and the necessity of relying on multiple protection barriers, including vaccination programs, high hygiene standards with proper personal protective equipment, and administration of antiviral surfaces, especially in areas of high human traffic [2]. Besides, some other viruses, including human norovirus (HuNV), can also attain tedious diverse transmission routes (via airborne vomitus dissemination, person-to-person and contaminated surfaces’ contact, food, and the environment) [3,4]. In addition, HuNV can remain infectious from two weeks to even as long as two months, depending on the environment [4]. Such tedious norovirus transmissions make cutting fomite-based infections a critical, reliable way of norovirus containment and prevention of hundreds of thousands of gastroenteritic infections around the world [5] and numerous deaths, especially in older adults (≥65 years) [3]. Another central issue is the absence of licensed vaccines for HuNV, even with the attempts of developing some vaccine candidates to HuNV [3].

Regarding preventive strategies, several uncertainties and factors hinder people’s compliance to vaccination. Lazarus and colleagues [6] surveyed the factors influencing the global acceptance of COVID-19 vaccination. They found that the trust in governments and the age are the leading players in the vaccination acceptance, with respondents from China and Poland reporting the highest (88.6%) and lowest (27.3%) responses, respectively. They tied the most increased responses from Asia to the strong trust in their governments. Besides, peoples aged 25 to 65 years were more accepting of vaccination than the age category of 18 to 24 years. This vaccine hesitancy is not a surprise, as the World Health Organization (WHO) has already pointed to the fact that from 2019 that vaccination refusal could constitute one of the 10 global health threats [7]. As such, vaccinations may not be relied upon as a stand-alone solution.

Antiviral surface coatings have been suggested as a novel approach to tackling the surface-mediated transmission of viral diseases. Such coatings could provide a permanent, continuously acting barrier to transmission, and some studies have already shown the efficacy of metallic compounds in reducing the infectivity of SARS-CoV-2 on surfaces [8,9].

Therefore, what nanomaterial tools could we have in stores to possibly combat the SARS-CoV-2, its variants, norovirus, or any viruses transmitted via surfaces? Assis et al. [10] demonstrated that SiO_2_ microparticles decorated with Ag nanoparticles (Ag NPs) within an ethyl vinyl acetate polymeric matrix inactivated SARS-CoV-2 by 99.26% and 99.62% after 2 and 10 min, respectively, following only two days of incubation. They attributed the SARS-CoV-2 inactivation to the induced reactive oxygen species (ROS; OH* and O_2_H*) at the interaction of SiO_2_ with H_2_O and O_2_. A recent work by Hosseini et al. [11] showed the reducing infectivity effect of a ZnO/SiO_2_ coating (water contact angle of 72° ± 3° and thickness of 30 ± 2 µm) on SARS-CoV-2 by a >99.98% reduction (>3 log reduction as detected by the median tissue culture infectious dose (TCID_50_)) after 1 h contact, recommending the administration of such coatings on handrails or doorknobs to reduce the spread and infectivity of SARS-CoV-2. They hypothesized that the reduced SARS-CoV-2 infectivity could be linked to two players as follows: the induced destructive ROS by Zn^2+^ ions released and the porosity of the hydrophilic coating, trapping the soaked viral droplet and allowing the facilitation of the ZnO antimicrobial role. Mantlo and colleagues [12] demonstrated the efficacy of copper ions (Cu^2+^) in Luminore CopperTouch (copper and copper–nickel) surfaces, producing ROS in the inactivation of SARS-CoV-2 by 99% after 2 h of contact and 99.9% of Ebola Marburg viruses after 30 min of contact through a hypothetic approach to viral genome disruption by the produced free radicals. In contrast to the previously mentioned role of Cu^2+^ in SARS-CoV-2 inactivation, Hewawaduge et al. [13] showed the inactivation properties of a solid-state copper sulfide (CuS)-incorporated three-layer mask (4.4%, 17.6%, and 0% CuS in the outer, middle, and inner layers of the mask, respectively) in reducing SARS-CoV-2 viral copies in a dose- and time-dependent manner. Besides, Hosseini et al. [14] demonstrated that a hydrophilic cupric oxide (CuO) coating almost entirely inactivated SARS-CoV-2 after 30 min and 1 h and attributed the viral inactivation mechanism to contact inactivation, depending on the rate of the viral transport to the active surface.

Concerning norovirus inactivation, Warnes and Keevil [15] also demonstrated the inactivation efficiency (with over a 4 log reduction) of copper ions (Cu(I) and Cu(II)) within copper and copper–nickel (containing 89% Cu) alloy surfaces on murine norovirus-1 (MNV-1, as a surrogate virus to HuNV) wet fomite contamination simulation within 30 and 60 min, respectively. Inactivation was even faster in dry fomite contamination of 1µL MNV. HuNV is very challenging to cultivate in cell cultures [16]. Therefore, surrogate viruses are used instead, and MNV currently remains the best surrogate, surpassing even the Tulane surrogate virus. The better performance of the surrogate MNV was attributed to its better persistence over broad pH range values and at 2 ppm of chlorine than Tulane virus, and while the MNV remained infectious (a titer of ~5 log PFU/mL) in tap water at 4 °C for over 30 days [17].

The importance and originality of this study are its simultaneous exploration of the inactivation efficacy of Cu–Ag nanocomposite surfaces on the Alpha variant (as a VOC) and MNV as a surrogate for HuNV (the prime causative agent of global viral gastroenteritis), with both transmitting through contaminated surfaces. We thoroughly investigated the physicochemical characteristics of the tested surfaces to draw conclusions on the main elements inactivating the Alpha variant and MNV. The aim was to find fruitful means for efficient applications of such inactivating surfaces in highly congested areas (e.g., schools, public transportations, and, most importantly, within health, hospital, and live-stock reservoirs), breaking the transmission chains of the pandemic and HuNV.

## 2. Materials and Methods

### 2.1. Copper –Silver Nanocomposite Surfaces and Their Characterization

**Figure 1:**
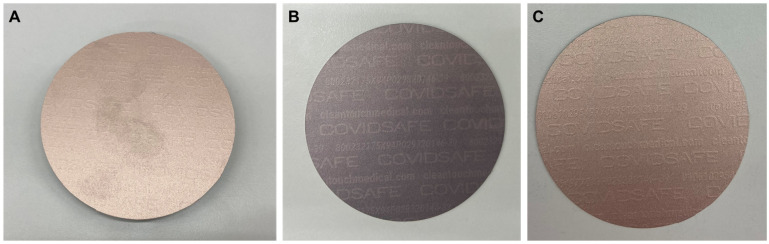
Different surface-plated samples with different colors were delivered to the University of Helsinki for inhibition testing against severe acute respiratory syndrome coronavirus 2 (SARS-CoV-2) (samples A and B) and murine norovirus (MNV) (sample C): (**A**,**C**) are bronze-brown surfaces, and (**B**) is dark brown surface. All surfaces were imprinted with COVIDSAFE labeling, representing surface roughness.

Three surface samples, namely A, B, and C, were provided by the Clean Touch Medical LTD to the University of Helsinki to experiment for the inhibition of the SARS-CoV-2 Alpha variant (B.1.1.7, Alpha variant) on 5 February 2021 and MNV (Figure 1). The company provided information that the samples were copper and silver hybrid combinations coated with an even 40 µm thickness on stainless-steel substrates. The coating was accomplished by wet painting using a spray for surface plating.

A scanning electron microscope was used to investigate the surface physicochemical properties (shape, size, and composition) of the samples provided and develop grounds for how viral inactivation could be achieved. The samples were placed on aluminum stubs and air-blown. Then, a scanning electron microscope (Zeiss Crossbeam 540, Zeiss, Germany) was operated using a secondary electron detector with a 15 kV acceleration voltage for imaging and energy-dispersive X-ray spectroscopy (EDX, collected in the mapping, high-quality mode with 100 frames and run for 1 h for each map). The Cu and Ag particles’ size distributions detected on the obtained SEM images were analyzed by ImageJ software (National Institutes of Health, Bethesda, MD, USA). Besides, the crystal structures of the Cu–Ag nanocomposite surfaces were measured by the XRD PANalytical X’pert Powder Pro diffractometer with Cu Kα radiation with a monochromator over a 30°-to-100° 2*θ* range. Surfaces were examined by an atomic force microscope (AFM; NanoWizard 4XP, JPK Bruker in the AC operation mode in the air using NCHV-A probes, Bruker, Santa Barbara, CA, USA) and the water contact angle (Attension Theta Flex optical tensiometer, Biolin Scientific, Gothenburg, Sweden) to detect their roughness and wettability, respectively.

### 2.2. SARS-CoV-2 and Norovirus Strains, Cell Cultures, and Surface Exposure Experiments

Cultured Vero E6 cells in minimal essential Eagle’s medium (MEM; Sigma-Aldrich, Saint Louis, MI, USA) supplemented with fetal bovine serum (FBS; Gibco; 10% for maintenance and 2% for infection), L-glutamine, penicillin, and streptomycin were infected with the SARS-CoV-2 Alpha variant (B.1.1.7, passage 1) and incubated at 37 ± 2 °C with 5% CO_2_. The cell media were collected, and using a plaque assay, the stock concentration was estimated to be 1 × 10^6^ PFU/mL. The coated surfaces (samples A and B) were first UV-disinfected in a UV chamber for 1 min. Then, 25 µL of SARS-CoV-2 viral suspension (1 × 10^6^ PFU/mL) were spread on both Cu –Ag nanocomposite-coated surfaces and glass surfaces that acted as a negative control surface. At time points of 1, 5, and 10 min, all surfaces were sampled with pre-wetted cotton swabs. Then, the swabs were placed in 500 µL MEM media, and from these, a 1:100 (*v*:*v*) viral dilution was prepared. Vero E6 cells in the 6-well plates were inoculated with 150 µL of the dilutions and incubated for 1 h at 37 °C. After the incubation, the cell media were replaced with fresh 2% MEM, and the cells were incubated for four days at 37 °C. An inoculation with viral suspensions and a clean MEM alone were used as positive and negative controls, respectively. Al cultures were performed in duplicate. The SARS-CoV-2 surface experiments were conducted in a registered BSL-3 facility at the Faculty of Veterinary Medicine, University of Helsinki.

The MNV stock contained approximately 1 × 10^6^ to 3 × 10^6^ (log_10_ 6) TCID_50_ units per milliliter, and the concentrated MNV contained approximately 1 × 10^7^ (log_10_ 7) TCID_50_ units per milliliter. The virus was propagated in the presence of 10% FBS (extra protein load). MNV (supplied by Prof. Virgin IV, University of Washington, USA) was cultivated in RAW 264.7 gamma NO (-) cells (mouse monocyte-macrophage line, ATCC^®^ CRL2278™). For TCID_50_ assay testing, 96-microwell plates in which 2.0 × 10^4^ RAW 264.7 cells per well in 10% DMEM (Dulbecco’s Modified Eagle Medium supplemented with 10% FBS) grew were prepared one day before the experiment. The coated surfaces (sample C) were tested, and a microscopic glass slide was used as a negative surface control. Before the test, the coated surfaces were cleaned with a mild detergent, rinsed with sterile distilled water and let dry. Viral aliquots of 1 µL (concentrated MNV) or 25 µL (non- concentrated MNV) were added onto the coated and control surfaces (a 3.1 cm^2^ area was delimited by a hot glue) for 1, 5, and 30 min. After the exposure time, the remaining virus was collected after washing the surfaces with 1 mL of 0% DMEM. Ten-fold dilutions of the samples were prepared in 0% DMEM. The amount of the remaining infectious virus was tested by cell culture. The results were given as TCID_50_. Virus log_10_-reduction on coated plates was calculated as compared to virus collected from control plates. One or two surface areas were tested for each sample and control in each experiment. One well per product was inoculated with 1 or 25 µL of 10% DMEM for cell cytotoxicity controls. For 25 µL inocula, it was ensured that the liquids were spread across the surface area. Three separate experiments were executed on different days.

### 2.3. Cytopathic Effects (CPEs) by the Alpha Variant and Microscopy of MNV-Infected Cells

After incubating the infected Vero E6 cells for 4 days, the 6-well plates were investigated under an inverted microscope (CKX41, Olympus Life Science Corporation, Tokyo, Japan) to detect the presence or absence of CPEs induced by the viable SARS-CoV-2 viruses. The CPE visualization was executed by crystal violet staining. The cells were fixed by adding 650 µL formaldehyde (37 wt%)/well for 30 min). Washing was performed with water (900 µL/well) followed by staining with crystal violet (625 µL of a 1:5 (*v*:*v*) diluted crystal violet solution for 10 min) and final washing with water (900 µL/well). Violet-stained cells represented viral inhibition, while clear wells indicated viable viruses infecting the cells. A digital camera was used to photograph the stained plates.

For the MNV, after making the sample dilutions, 200 µL of cell culture media were removed from the 96-well cell culture plates before adding 100 µL original and diluted samples per well. Each sample, original or diluted, was tested in 6 parallel wells. The cell culture plates were incubated in a cell cabinet at 37 °C and with 5% CO_2_ for 1 h. The inoculates were removed and replaced by 200 µL of 10% DMEM per well. The cell culture plates were further incubated in a cell cabinet at 37 °C and with 5% CO_2_ for 1 week. The plates were inspected by a microscope daily, documenting the results as positive or negative, and the final reading was conducted on the 7th day after inoculation. The TCID_50_/mL value was obtained using a TCID_50_ calculator (as previously reported [18] using the Spearman–Kärber algorithm). Average values and standard deviations were calculated using Microsoft Excel. Log reduction was calculated from the TCID_50_ log_10_ value of the virus control (without the coating) made in the same test occasion subtracted by the TCID_50_ log_10_ value from a sample from the coated surface.

### 2.4. RT-PCR of the Tested Alpha Variant Samples

Following the Vero E6 cells 4-day incubation, aliquots of the cell culture media were transferred to new plates for RNA isolation and further RT-qPCR to ensure that the reported CPE was exclusively induced by the SARS-CoV-2.

RNA extraction was performed by QIAcube HT (Qiagen, Hilden, Germany) using a QIAamp 96 Virus QIAcube HT kit (Qiagen, Hilden, Germany), adopting the kit protocol after off-board lysis in BSL-3. The protocol was executed as follows: (i) 200 µL of the sample were added to 160 µL of ACL lysis buffer with carrier RNA and 20 µL of Proteinase K in BSL-3, and the mixed solution was incubated for 30 min at room temperature. (ii) The lysis blocks surfaces containing the lysed samples were wiped and disinfected with 80% ethanol and transferred outside of BSL-3 to continue the isolation protocol. (iii) RT-qPCR targeting the SARS-CoV-2 N gene was performed as described by Corman et al. [19]. Samples were reported positive for SARS-CoV-2 infections with reference to the cycle threshold (Ct) values. When the reported Ct values were >30, it indicated either the absence of viral particles or the inhibition of infectious SARS-CoV-2 particles growth, with all viral RNA coming from the initial inoculum.

## 3. Results and Discussion

### 3.1. Copper–Silver Nanocomposite Surfaces and Their Characterization

**Figure 2:**
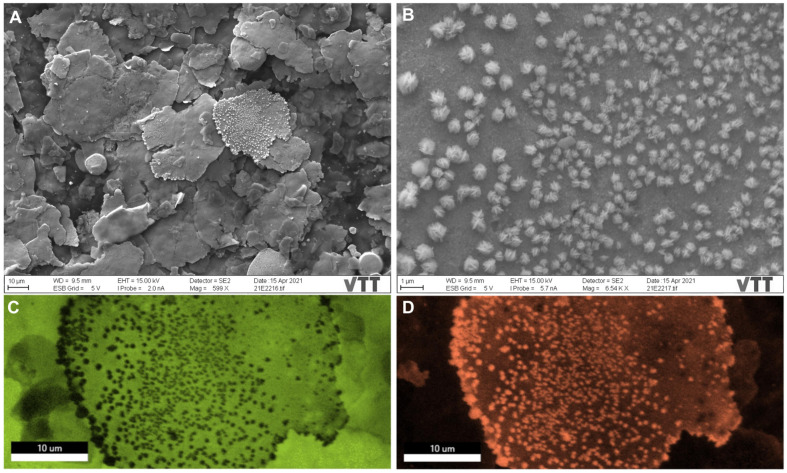
SEM images and energy-dispersive X-ray spectroscopy (EDX) mapping of sample A. (**A**) SEM image of morphological irregular copper flakes. (**B**) SEM image of star-shaped Ag nanoparticles (NPs) decorating the copper flakes. (**C**) Mapped image of morphological irregular copper flakes denoted in green. (**D**) Mapped image of star-shaped Ag NPs decorating the copper flakes denoted in orange.

**Figure 3:**
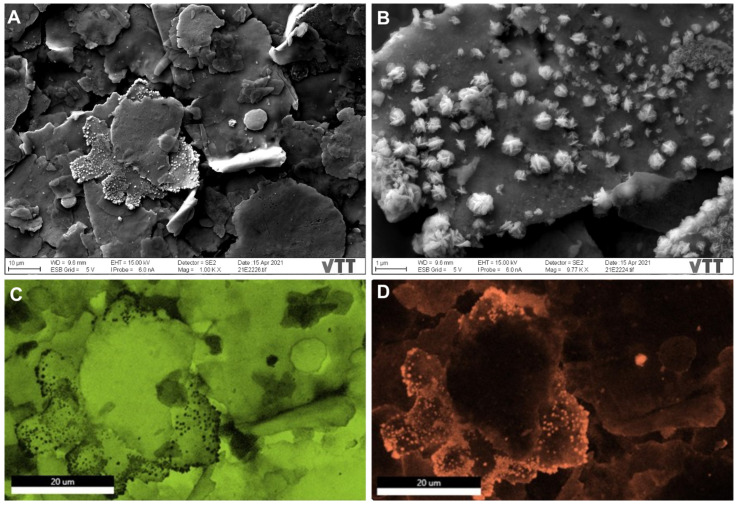
SEM images and EDX mapping of sample B. (**A**) SEM image of morphological irregular copper flakes. (**B**) SEM image of star-shaped Ag NPs decorating the copper flakes that were more branching than sample A. (**C**) Mapped image of morphological irregular copper flakes denoted in green. (**D**) Mapped image of star-shaped Ag NPs decorating the copper flakes denoted in orange.

**Figure 4:**
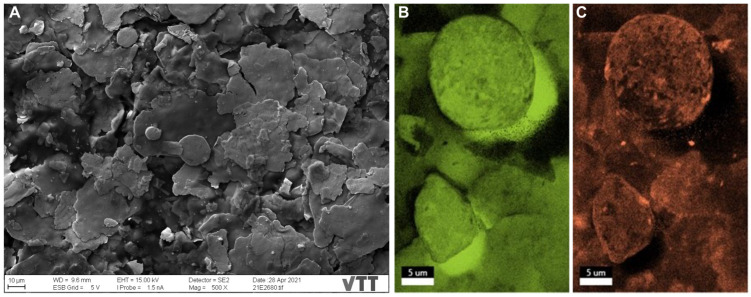
SEM images and EDX mapping of sample C. (**A**) SEM image of morphological irregular copper flakes. (**B**) Mapped image of morphological irregular copper flakes denoted in green. (**C**) Mapped image of some Cu particles displayed as the spherical-shaped and irregular pear-shaped particles doping irregular spherical Ag NPs denoted in orange.

The surface of sample A was not entirely clean even after its air blowing for SEM investigations (Figure 1A). SEM imaging and EDX analyses demonstrated the surface morphology and the chemical structure of the samples. Sample A (Figure 2A) shows the different morphologies of the Cu particles fluctuating from large irregular flakes to rounded, spherical-shaped particles (10.3 ± 0.9 µm in size). Some copper flakes were decorated by porous star-shaped Ag particles (Figure 2B) with a 613 ± 186 nm size. Spherical-shaped copper particles (4.1 ± 1.6 µm in size) are noted in Figure 3 for sample B with star-shaped Ag particles that were more branching of size (902 ± 449 nm), giving a flower-shaped morphology. Regarding sample C, different morphologies were observed with the copper particles taking both spherical-shaped and irregular pear-shaped morphologies (8.9 ± 3.9 µm in size) with irregular spherical Ag NPs (1.6 ± 0.6 µm in size) embedded within the copper particles. All particles were measured from a sum of 10 particles. Comparing these results with those obtained in our previous study for surfaces provided by the same manufacturer and inhibited SARS-CoV-2 Wuhan reference strain [20], the present samples (A, B, and C) possessed smaller copper particles and much larger Ag particles. Besides, the star-shaped and flower-shaped morphologies of Ag NPs in samples A and B were not reported in our same previous work [20]. The Cu and Ag distributions within the mapped areas (Figure 2, Figure 3 and Figure 4) are denoted by fluorescent green and orange colors, respectively (Figure 2C,D). Tables S1–S3 delineate the EDX quantitative chemical compositions of samples A, B, and C, respectively. The highest concentrations were always reserved for Cu as ~56, ~59, and ~48 wt% for samples A, B, and C, respectively. Correspondingly, the second highest metal concentrations kept for Ag were ~28, ~13, and ~11 wt% for samples A, B, and C, respectively. Other elements detected were represented in smaller variable quantities in a descending manner as follows (C, O, Sn, Al, and Si). Similar to the size comparison with our previous work, the present elemental composition contained a less Cu content and a more Ag content in the three samples tested in the present study for Alpha variant inhibition compared with in the previously tested samples for the Wuhan strain [20].

**Figure 5:**
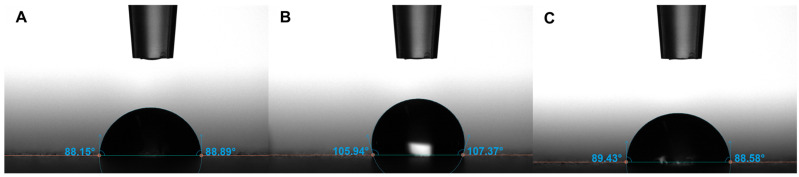
(**A**–**C**) Water contact angle measurements of surfaces.

Figure S1 displays XRD patterns of Cu–Ag nanocomposites. The Cu and Ag peaks were indexed with reference patterns of ICDD-00-003-1005 for Cu and ICDD 00-003-1005 for Ag, respectively. The sharp dominant peaks of Cu indicated large crystalline Cu particles, and broad small peaks of Ag indicated its nanocrystal size nature. The AFM revealed that all surfaces (A, B, and C) were very rough with root mean square roughness values of 1291, 983, and 1602 nm, respectively, and their images are demonstrated in Figure S2. The water contact angle measurements of surfaces A, B, and C are shown in Figure 5, detecting surfaces’ wettability and displaying more hydrophobicity of surface B than those of surfaces A and C.

### 3.2. Surface Inhibition of the Alpha Variant and MNV

**Figure 6:**
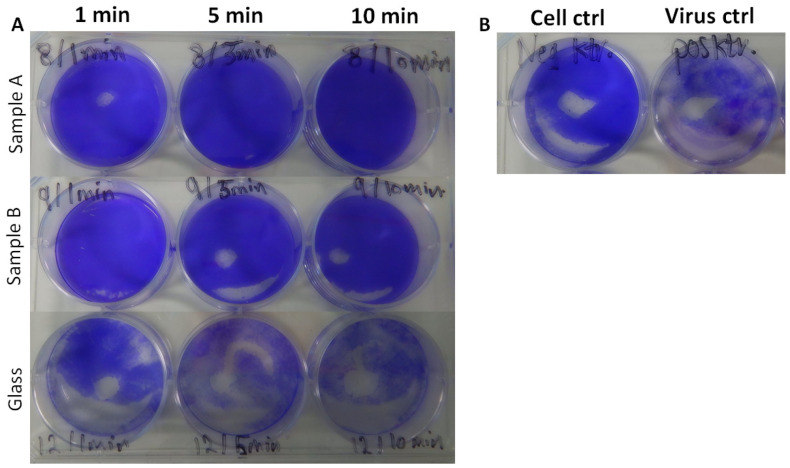
(**A**) Image demonstrating the inhibition of the Alpha variant growth on Cu–Ag nanocomposite-plated surfaces (samples A and B) after 1, 5, and 10 min, based on crystal violet-stained Vero E6 cells. (**B**) Image of cells without the virus used as a negative control and viral cultures without the surface treatment used as a positive control. The blank color shows cell death caused by, for example, viral growth, cells’ aging, or mechanical stressors), and the deep violet color demonstrates an intact cell layer.

**Figure 7:**
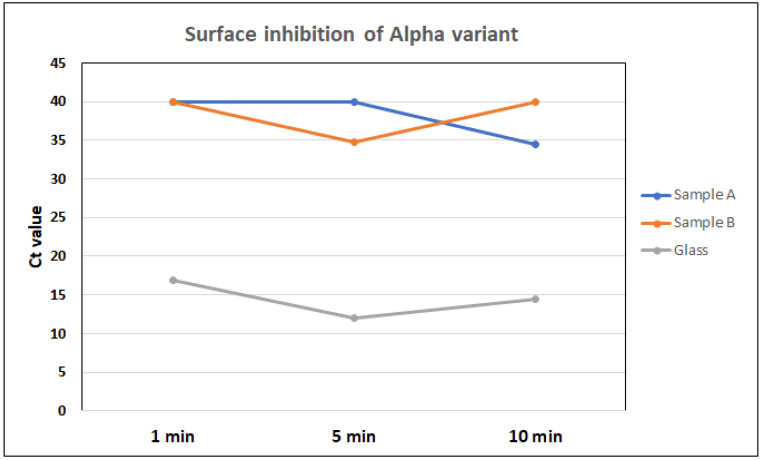
Ct values of the RT-PCR that were performed from post-culture media of separate wells. Ct values were set to 40 for samples with no Ct values.

No CPEs were detected in culture samples collected from surfaces A and B based on microscopic observations. However, CPEs were detected in samples collected from glass (Figure 6 and Table S4). To exclude any other role inducing CPEs than SARS-CoV-2, virus cultures were tested with PCR (Figure 7 and Table S4). The Ct values of glass samples are under 20, whereas values from surface A and B samples are over 30 in all cases. This showed that there was SARS-CoV-2 growth in all the samples from glass, but no signs were seen in samples from surfaces A and B. High Ct values surpassing 30 and the lack of a visible CPE indicated that SARS-CoV-2 was inhibited to undetectable levels very fast, in less than 1 min, on surfaces A and B. Ct values over 30 were considered to originate from the original viral inoculum and were not from infectious viral particles. A small amount of infectious virus on surfaces A or B during sampling could not be entirely excluded. However, we used a high virus amount in the experimental settings, and the amount of infectious viral particles on surfaces could be expected to be much lower. As the viral titer decreased overtime on all surfaces, some speculations could refer that the inactivation was not entirely attributed to the nanocomposites, but also from the substrates used. Based on the information provided by the manufacturer, the substrates used for the plated surfaces were formed from stainless steel. The surface coating of the nanocomposite materials formed 40 µm uniform-thickness layers over the substrates. However, earlier studies on SARS-CoV-2 stability on surfaces within laboratory settings indicated no significant reduction in viral titers on stainless steel or glass during 10 min [21]. Moreover, SARS-CoV-2 has shown stability on stainless steel surfaces at extended time points, reaching from three [21], four [22] to seven days [23]. Bonil et al. [24] also demonstrated the stability of SARS-CoV-2 on both stainless steel and glass for several hours with viral half-lives of 6.9 and 3.5 h, respectively. In summary, the interesting comparison of SARS-CoV-2 inactivation results from surfaces A and B with that from glass surfaces obviously demonstrated the efficient Alpha variant inhibition in less than 1 min. Furthermore, in order to confirm the same notion of the stability of MNV on stainless steel, we ran a preliminary investigation using steel as a negative control, and no reduction in the infectious MNV amount on steel was observed even after 30 min (Figure S3).

**Figure 8:**
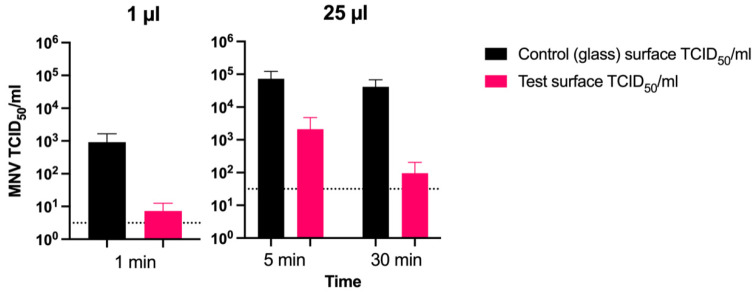
Median tissue culture infectious dose (TCID_50_) values of MNV dilutions after the surface exposure to the Cu–Ag nanocomposite surface (sample C) compared with that to the negative control glass. MNV showed log_10_ reductions of 2.10 (±0.27), 1.69 (±0.40), and 2.72 (±0.40) after 1, 5, and 30 min, respectively, in comparison with on the glass surface. Dashed lines indicate limits of detection.

After 1 min exposure, using a small 1 µL volume of concentrated MNV, an average log_10_ reduction of 2.10 (±0.3) was observed on the coated surface C compared with on a control negative glass surface (Figure 8). Once out of 5 trials (3 experiments), no leftover virus was demonstrated on the surfaces exposed. Following a 5 min exposure with a higher viral aliquot (25 µL), the number of infectious MNV was reduced by an average log_10_ reduction of 1.7 (±0.4). After a longer 30 min exposure with a 25 µL viral aliquot, the number of MNV was reduced by an average log_10_ reduction of 2.7 (±0.4) in comparison with on the control negative glass surface that showed almost no reduction in MNV infectivity.

Our results showed that the number of infectious MNV reduced faster on the coated surface (sample C) than on the glass surface. The decrease rate was approximately 1 log_10_ unit on the coated surface C (vs. 0.2 log_10_ units on the glass surface; Figure 8) from 5 to 30 min. The viral inactivation rate on surfaces was mainly influenced by the length of time and the viral amount added to the surface. Therefore, a rapid effect of 2.1 log_10_ unit decrease even after 1 min was demonstrated, when fewer infectious viruses were added on the coated surface C. This result is supported by previous research results of Warnes and Keevil [15], although the coated copper-containing surfaces were not identical typically within the compared studies. Other influencing factors, such as the surface porosity and hydrophobicity, relative humidity, temperature, and viral drying on surfaces, also generally affect the viral persistence of viruses (in general and in particular SARS-CoV-1 and -2) with a longer viral persistence on less porous, more hydrophobic surfaces and around room temperature with a humidity of around 40% [2]. Collectively, the present results point towards well-induced inactivation properties of such nanocomposite surfaces against both MNV and the Alpha variant after even only one minute, promoting their surface administration not only in high traffic public and hospital settings, but also on the handles of public toilets. This toilet surface administration stems from the fact that HuNV is a critical gastrointestinal pathogen that can infect with a small viral inoculum of <100 viral particles [25], and SARS-CoV-2 can remain viable in human feces from 2 h to 2 days and even in human urine from 3 to 4 days. Therefore, the present inactivation surfaces also provide an attractive, promising opportunity to cut the gastrointestinal transmission of HuNV and fecal–oral transmission of SARS-CoV-2 [23].

When it comes to the downside of the anti-SARS-CoV-2 nanomaterial role, two issues fuel the challenging administration of such antiviral nanosystems. Firstly, the toxicity concerns associated with the application of anti-coronavirus nanosystems should be weighted carefully regarding the different unstandardized physicochemical parameters of such nanosystems. Such diverse physicochemical properties are, unfortunately, understudied for their toxicity in different settings, e.g., nano-bio interfaces (interaction of nanoparticle surfaces and biological components with the formed protein corona), in vitro, and in vivo [26]. Secondly, the antiviral mechanistic mode of actions of such nanosystems in specifically SARS-CoV-2 and its variants remains ambiguous because of the novelty of the SARS-CoV-2 pandemic and mutations of its variants, leading many researchers to attribute the anti-SARS-CoV-2 of many nanosystems, e.g., sub-micrometer ZnO/SiO_2_ coatings [11], to their antibacterial effects of the released metal ions attracting negatively charged bacterial groups, causing an ultimate destruction of bacterial membranes and cells [27]. The lack of sufficient data explaining anti-SARS-CoV-2 inhibition mechanistic actions could explain these antiviral properties attribution to the antibacterial ones.

Hewawaduge and colleagues [13] suggested a mechanistic SARS-CoV-2 inactivation role of the solid-state CuS to the caused damage of the viral envelope, hindering their ability to infect host cells. Therefore, they suggested its antiviral role was only played in enveloped viruses (e.g., coronaviruses or influenza) due to the absence of any antiviral activity when CuS was tested against the non-enveloped *Salmonella* phage P22. However, this contradicts our results of Cu–Ag nanocomposite surfaces inactivating MNV, a non-enveloped positive-stranded RNA virus [25]. When it comes to even the mechanistic time frame of inactivation, the predefined time points of incubation and infection considerably vary among different studies. Hosseini and collaborators [14] reported the inactivation time of SARS-CoV-2 to be after 30 min and 1 h, because the mechanism of their inactivation was attributed to the viral transport to the coating surface, adopting a contact inactivation, which could take a longer time because viral diffusion needs a longer time than ionic diffusion due to the larger radius viral size than that of metal ions. According to our best knowledge, the lowest time point of inactivation and a decrease in viral load were reported in the present study after 1 min for Cu–Ag nanocomposites against the Alpha variant and MNV, respectively. Furthermore, as our surfaces are inclined to be hydrophobic, then most likely, the inactivation mechanism is attributed to the dissolved ions from the surfaces explaining the swift time of inactivation in the present study (after 1 min). On the contrary, this inactivation time of Cu ions in previous studies fluctuates from the lowest inactivation points of 1 h and 30 min for SARS-CoV-2 [12] and MNV [15], respectively. In the present work, sample B was more hydrophobic than sample A based on water contact angle measurements (Figure 5). Therefore, even though the anti-SARS-CoV-2 inhibitions by both samples were almost similar and swifted after only 1 min, the more hydrophobicity of sample B could facilitate the release of more metal ions, which would be an exciting point that needs further investigations to be confirmed. Ultimately, to highlight the key role of the elicited antiviral properties in light of the spotted hypothesis of the released ions, inactivating the viruses on surfaces, we presented the following argumentation. The Cu/Ag ratio in the present work was much less than the Cu/Ag ratio in our previously published work for surfaces inhibiting the SARS-CoV-2 Wuhan reference strain at 1 min. However, the present copper particles were smaller than previous ones [20]. Bezza et al. [28] showed that the more cuprous ion release facilitated more antimicroibal effects, based on the fact that smaller particles could release more ions allowing more antimicrobial properties [29]. Therefore, we could attribute the present surface antiviral properties to firstly the dominating copper element, releasing ions that inactivate viral particles and smaller copper particles that were preferable to release more ions, exhibiting more antiviral properties.

The contribution of this study is to demonstrate the efficacy and swift the surface inactivation of the SARS-CoV-2 Alpha variant and MNV after only 1 min, cutting their tedious transmission routes and providing a means for their surface administration in highly congested areas (e.g., schools, public transportations, public toilets, and hospital and live-stock reservoirs).

## 4. Conclusions

The present work shows a fast inactivation of the SARS-CoV-2 Alpha variant and an apparent reduction in the MNV load after only one minute of contact to surfaces containing Cu–Ag nanocomposites with the dominance of the Cu element (~56, ~59, and ~48 wt%) combined with the Ag compositions (~28, ~13, and ~11 wt%, respectively) within the tested three different surfaces. We recommend applying such inhibitory surfaces to break the SARS-CoV-2 and HuNV challenging transmission routes at high traffic places, including schools, public transportations and toilets, and hospital and live-stock reservoirs. There is still considerable room for enhancement before the practical application of such surfaces, including a thorough in vitro and in vivo toxicity assessment of both nanocomposite formulations and surfaces while standardizing physicochemical parameters, testing protocols, and animal models used for the assessment.

## Data Availability

The data presented in this study are available on request from corresponding authors.

## Supplementary Materials

The following supporting information can be downloaded at: https://www.mdpi.com/article/10.3390/nano12071037/s1, Table S1: EDX quantitative chemical composition of sample A in a concentration-descending manner. Table S2: EDX quantitative chemical composition of sample B in a concentration-descending manner. Table S3: EDX quantitative chemical composition of sample C in a concentration-descending manner. Figure S1. XRD patterns of Cu–Ag nanocomposite surfaces using Cu Kα radiation over a 30°-to-100° 2*θ* range with blue and green lines indicating Cu and Ag diffraction peaks with ICDD reference patterns for Cu and Ag, respectively. Figure S2. AFM amplitude error images showing the rough surfaces of samples A, B, and C. Table S4: Culture results expressed as the presence of cytopathic effects (CPEs) on cells and Ct values of the RT-PCR performed on post-culture media. Figure S3. A logarithmic scale (10,000 equates log_4_ TCID_50_) showing no reduction in the infectious MNV amount on stainless steel (as a negative control) after 30 min in comparison with the observed tremendous viral reduction on the tested surface (sample C) in a preliminary investigation.

## References

[1] WHO (2022). COVID-19 Weekly Epidemiological Update.

[2] Rakowska P.D., Tiddia M., Faruqui N., Bankier C., Pei Y., Pollard A.J., Zhang J., Gilmore I.S. (2021). Antiviral Surfaces and Coatings and Their Mechanisms of Action. Commun. Mater..

[3] Cardemil C.V., Parashar U.D., Hall A.J. (2017). Norovirus Infection in Older Adults: Epidemiology, Risk Factors, and Opportunities for Prevention and Control. Infect. Dis. Clin. N. Am..

[4] Lopman B., Gastañaduy P., Park G.W., Hall A.J., Parashar U.D., Vinjé J. (2012). Environmental Transmission of Norovirus Gastroenteritis. Curr. Opin. Virol..

[5] Ahmed S.M., Hall A.J., Robinson A.E., Verhoef L., Premkumar P., Parashar U.D., Koopmans M., Lopman B.A. (2014). Global Prevalence of Norovirus in Cases of Gastroenteritis: A Systematic Review and Meta-Analysis. Lancet Infect. Dis..

[6] Lazarus J.V., Ratzan S.C., Palayew A., Gostin L.O., Larson H.J., Rabin K., Kimball S., El-Mohandes A. (2021). A Global Survey of Potential Acceptance of a COVID-19 Vaccine. Nat. Med..

[7] World Health Organiztion Ten Threats to Global Health in 2019. https://www.who.int/news-room/spotlight/ten-threats-to-global-health-in-2019.

[8] Behzadinasab S., Chin A., Hosseini M., Poon L., Ducker W.A. (2020). A Surface Coating That Rapidly Inactivates SARS-CoV-2. ACS Appl. Mater. Interfaces.

[9] Mlcochova P., Chadha A., Hesselhoj T., Fraternali F., Ramsden J.J., Gupta R.K. (2020). Extended In Vitro Inactivation of SARS-CoV-2 by Titanium Dioxide Surface Coating. bioRxiv.

[10] Assis M., Simoes L.G.P., Tremiliosi G.C., Coelho D., Minozzi D.T., Santos R.I., Vilela D.C.B., do Santos J.R., Ribeiro L.K., Rosa I.L.V. (2021). SiO_2_-Ag Composite as a Highly Virucidal Material: A Roadmap That Rapidly Eliminates SARS-CoV-2. Nanomaterials.

[11] Hosseini M., Behzadinasab S., Chin A.W.H., Poon L.L.M., Ducker W.A. (2021). Reduction of Infectivity of SARS-CoV-2 by Zinc Oxide Coatings. ACS Biomater. Sci. Eng..

[12] Mantlo E.K., Paessler S., Seregin A., Mitchell A. (2021). Luminore CopperTouch Surface Coating Effectively Inactivates SARS-CoV-2, Ebola Virus, and Marburg Virus in Vitro. Antimicrob. Agents Chemother..

[13] Hewawaduge C., Senevirathne A., Jawalagatti V., Kim J.W., Lee J.H. (2021). Copper-Impregnated Three-Layer Mask Efficiently Inactivates SARS-CoV-2. Environ. Res..

[14] Hosseini M., Chin A.W.H., Behzadinasab S., Poon L.L.M., Ducker W.A. (2021). Cupric Oxide Coating That Rapidly Reduces Infection by SARS-CoV-2 via Solids. ACS Appl. Mater. Interfaces.

[15] Warnes S.L., Keevil C.W. (2013). Inactivation of Norovirus on Dry Copper Alloy Surfaces. PLoS ONE.

[16] Ettayebi K., Tenge V.R., Cortes-Penfield N.W., Crawford S.E., Neill F.H., Zeng X.-L., Yu X., Ayyar B.V., Burrin D., Ramani S. (2021). New Insights and Enhanced Human Norovirus Cultivation in Human Intestinal Enteroids. mSphere.

[17] Hirneisen K.A., Kniel K.E. (2013). Comparing Human Norovirus Surrogates: Murine Norovirus and Tulane Virus. J. Food Prot..

[18] Hierholzer J.C., Killington R.A. (1996). Virus Isolation and Quantitation. Virol. Methods Man..

[19] Corman V.M., Landt O., Kaiser M., Molenkamp R., Meijer A., Chu D.K.W., Bleicker T., Brünink S., Schneider J., Schmidt M.L. (2020). Detection of 2019 Novel Coronavirus (2019-NCoV) by Real-Time RT-PCR. Eurosurveillance.

[20] Mosselhy D.A., Kareinen L., Kivistö I., Aaltonen K., Virtanen J., Ge Y., Sironen T. (2021). Copper-Silver Nanohybrids : SARS-CoV-2 Inhibitory Surfaces. Nanomaterials.

[21] van Doremalen N., Bushmaker T., Morris D.H., Holbrook M.G., Gamble A., Williamson B.N., Tamin A., Harcourt J.L., Thornburg N.J., Gerber S.I. (2020). Aerosol and Surface Stability of SARS-CoV-2 as Compared with SARS-CoV-1. N. Engl. J. Med..

[22] Chin A.W.H., Chu J.T.S., Perera M.R.A., Hui K.P.Y., Yen H.-L., Chan M.C.W., Peiris M., Poon L.L.M. (2020). Stability of SARS-CoV-2 in Different Environmental Conditions. Lancet Microbe.

[23] Liu Y., Li T., Deng Y., Liu S., Zhang D., Li H., Wang X., Jia L., Han J., Bei Z. (2021). Stability of SARS-CoV-2 on Environmental Surfaces and in Human Excreta. J. Hosp. Infect..

[24] Bonil L., Lingas G., Coupeau D., Lucet J.C., Guedj J., Visseaux B., Muylkens B. (2021). Survival of SARS-CoV-2 on Non-Porous Materials in an Experimental Setting Representative of Fomites. Coatings.

[25] Robilotti E., Deresinski S., Pinsky B.A. (2015). Norovirus. Clin. Microbiol. Rev..

[26] Mosselhy D.A., Virtanen J., Kant R., He W., Elbahri M., Sironen T. (2021). COVID-19 Pandemic: What about the Safety of Anti-Coronavirus Nanoparticles?. Nanomaterials.

[27] Mosselhy D.A., Granbohm H., Hynönen U., Ge Y., Palva A., Nordström K., Hannula S.-P. (2017). Nanosilver–Silica Composite: Prolonged Antibacterial Effects and Bacterial Interaction Mechanisms for Wound Dressings. Nanomaterials.

[28] Bezza F.A., Tichapondwa S.M., Chirwa E.M.N. (2020). Fabrication of Monodispersed Copper Oxide Nanoparticles with Potential Application as Antimicrobial Agents. Sci. Rep..

[29] Mosselhy D.A., Assad M., Sironen T., Elbahri M. (2021). Nanotheranostics: A Possible Solution for Drug-Resistant *Staphylococcus aureus* and Their Biofilms?. Nanomaterials.

