# Supplementary material for: Inhibition of SARS-CoV-2 Alpha Variant and Murine Noroviruses on Copper-Silver Nanocomposite Surfaces
Source: Nanomaterials (Basel). 2022 Mar 22;12(7):1037. doi: 10.3390/nano12071037 (PMC9000483; doi:10.3390/nano12071037)
Supplement: Supplementary file 1 [file nanomaterials-12-01037-s001.zip › nanomaterials-1619532-proof-SI-2.pdf]

Supplementary Materials

Inhibition of SARS-CoV-2 Alpha Variant and Murine Noroviruses on Copper-Silver Nanocomposite Surfaces

Dina A. Mosselhy [1,2,*], Lauri Kareinen [1,2], Ilkka Kivistö [1,2], Jenni Virtanen [1,2], Emil Loikkanen [3], Yanling Ge [4], Leena Maunula [3] and Tarja Sironen [1,2,*]

[1] Department of Virology, Faculty of Medicine, University of Helsinki, 00014 Helsinki, Finland; lauri.kareinen@helsinki.fi (L.K.); ilkka.kivisto@helsinki.fi (I.K.); jenni.me.virtanen@helsinki.fi (J.V.)

[2] Department of Veterinary Biosciences, Faculty of Veterinary Medicine, University of Helsinki, 00014 Helsinki, Finland

[3] Department of Food Hygiene and Environmental Health, Faculty of Veterinary Medicine, University of Helsinki, 00014 Helsinki, Finland; emil.loikkanen@helsinki.fi (E.L.); leena.maunula@helsinki.fi (L.M.)

[4] VTT Technical Research Center of Finland Ltd., 02044 Espoo, Finland; yanling.ge@vtt.fi

* Correspondence: dina.mosselhy@helsinki.fi (D.A.M.); tarja.sironen@helsinki.fi (T.S.)

Table S1. EDX quantitative chemical composition of sample A in a concentration descending manner.

Element	Weight (wt)%	Atomic %	Error %
**Cu K**	56	38.6	1.6
**Ag L**	27.8	11.3	1.1
**C K**	9.3	34	15.1
**O K**	4.5	12.4	15.1
**Al K**	1.2	2	1.8
**Sn L**	1.2	0.4	4.6
**Si K**	1	1.6	1.9

Table S2. EDX quantitative chemical composition of sample B in a concentration descending manner.

Element	Weight (wt)%	Atomic %	Error %
**Cu K**	59.1	30	3
**C K**	20.9	56.2	8.5
**Ag L**	12.7	3.8	1.6
**O K**	3.9	7.8	8.6
**Sn L**	2	0.6	2.7
**Al K**	0.8	0.9	7.9
**Si K**	0.7	0.8	6.9

Table S3. EDX quantitative chemical composition of sample C in a concentration descending manner.

Element	Weight (wt)%	Atomic %	Error %
**Cu K**	48.2	19.8	3
**C K**	30.9	67.1	8.2
**Ag L**	11.4	2.8	1.9
**O K**	4.7	7.7	9
**Sn L**	2.6	0.6	3.3
**Al K**	1.1	1	6.9
**Si K**	1	1	5.9

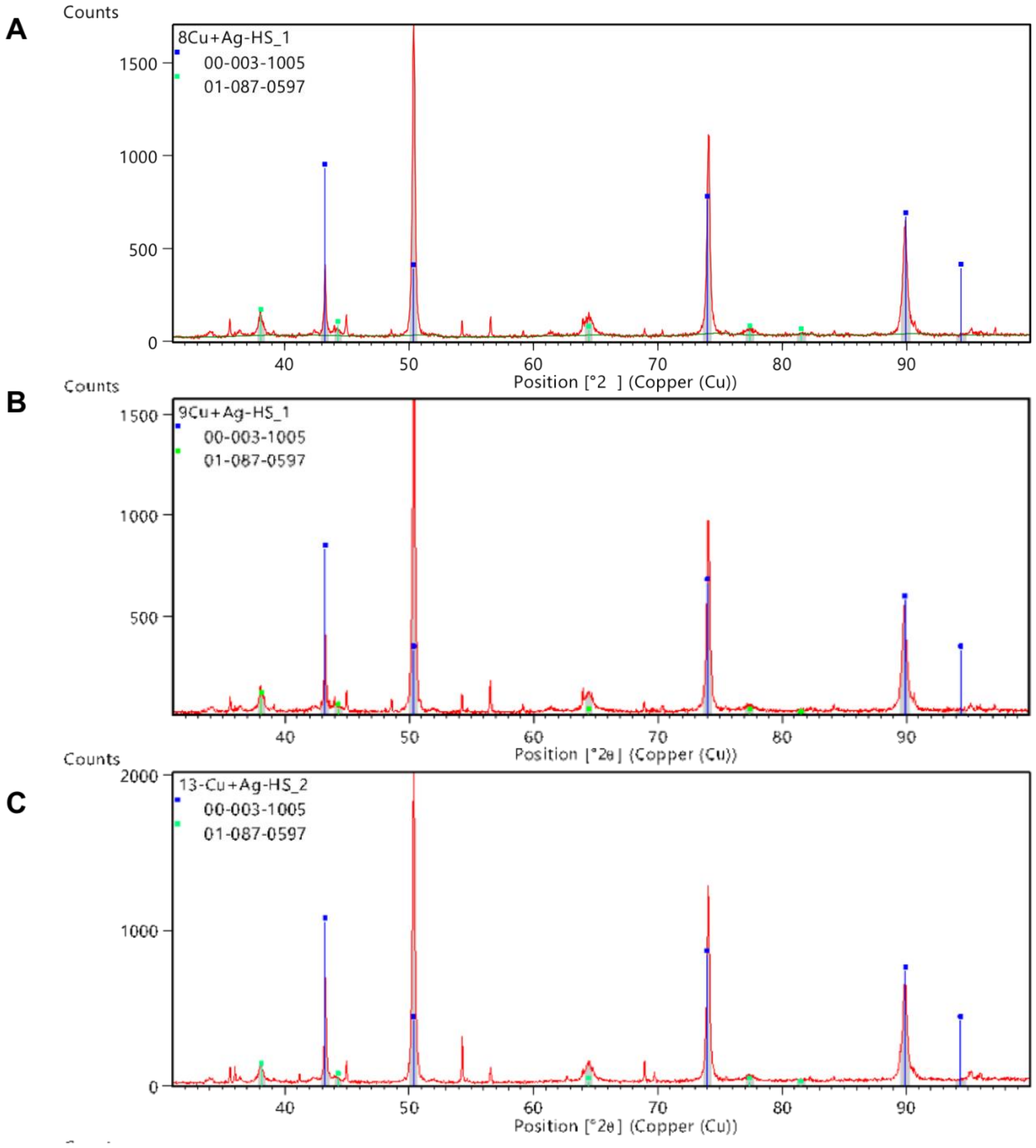

Figure S1. XRD patterns of Cu-Ag nanocomposite surfaces using Cu Kα radiation over a 30◦ to 100◦ 2θ range with blue and green lines indicating Cu and Ag diffraction peaks with ICDD reference patterns for Cu and Ag, respectively.

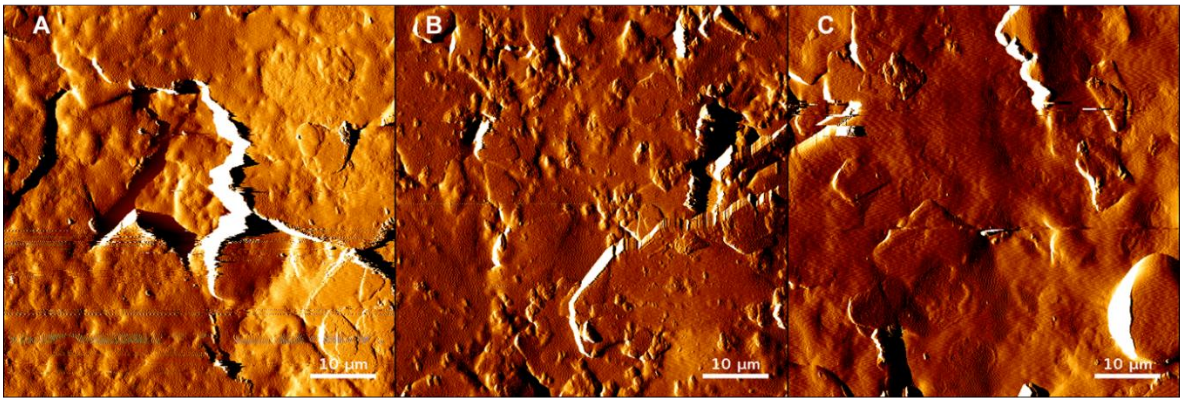

Figure S2. AFM amplitude error images showing the rough surfaces of samples A, B, and C.

Table S4. Culture results expressed as the presence of cytopathic effects (CPE) on cells and Ct values of the RT-PCR performed on post-culture media.

**-**	**1 min**		**5 min**		**10 min**	
**-**	**CPE**	**Ct values**	CPE	Ct values	CPE	Ct valuess
**Sample A**	+	No Ct	-	No Ct	-	34.52
**Sample B**	-	No Ct	+	34.82	+	No Ct
**Glass**	+++	16.87	+++	12.01	+++	14.50

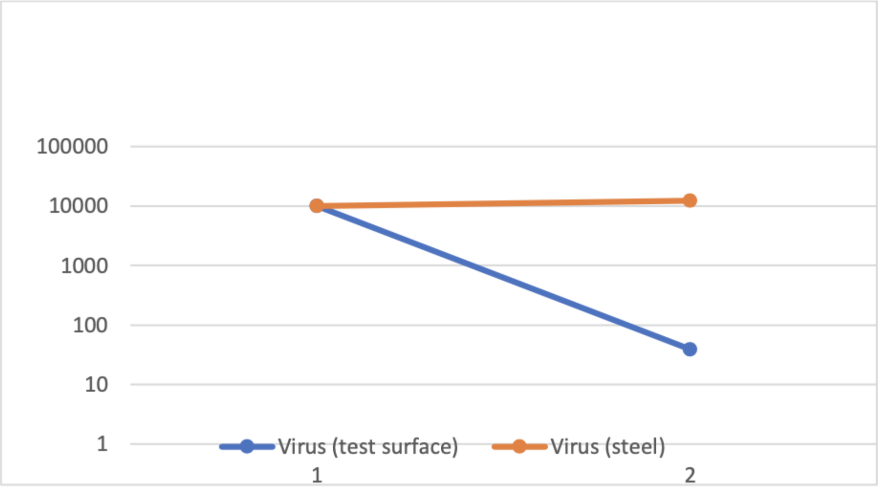

Figure S3. A logarithmic scale (10 000 equates log_4 $TCID_{50}$) showing no reduction in infectious murine norovirus (MNV) amount on stainless steel (as a negative control) after 30 min in comparison with the observed tremendous viral reduction on the tested surface (sample C) in a preliminary investigation.
